# The *Tribolium castaneum* cell line TcA: a new tool kit for cell biology

**DOI:** 10.1038/srep06840

**Published:** 2014-10-30

**Authors:** Kristopher Silver, Hongbo Jiang, Jinping Fu, Thomas W. Phillips, Richard W. Beeman, Yoonseong Park

**Affiliations:** 1 Department of Anatomy and Physiology, Kansas State University, Manhattan, KS 66506; 2 Department of Entomology, Kansas State University, Manhattan, KS 66506; 3 Key Laboratory of Entomology and Pest Control Engineering, College of Plant Protection, Southwest University, Chongqing 400715; 4 USDA, Agricultural Research Service, Center for Grain and Animal Health Research, 1515 College Ave, Manhattan, KS 66502

## Abstract

The red flour beetle, *Tribolium castaneum*, is an agriculturally important insect pest that has been widely used as a model organism. Recently, an adherent cell line (BCIRL-TcA-CLG1 or TcA) was developed from late pupae of the red flour beetle. Next generation transcriptome sequencing of TcA cells demonstrated expression of a wide variety of genes associated with specialized functions in chitin metabolism, immune responses and cellular and systemic RNAi pathways. Accordingly, we evaluated the sensitivity of TcA cells to dsRNA to initiate an RNAi response. TcA cells were highly sensitive to minute amounts of dsRNA, with a minimum effective dose of 100 pg/mL resulting in significant suppression of gene expression. We have also developed a plasmid containing two TcA-specific promoters, the promoter from the 40S ribosomal protein subunit (TC006550) and a bi-directional heat shock promoter (TcHS70) from the intergenic space between heat shock proteins 68a and b. These promoters have been employed to provide high levels of either constitutive (TC006550) or inducible (TcHS70) gene expression of the reporter proteins. Our results show that the TcA cell line, with its sensitivity to RNAi and functional TcA-specific promoters, is an invaluable resource for studying basic molecular and physiological questions.

The red flour beetle, *Tribolium castaneum*, is an economically important insect pest that has been widely used as a model organism in laboratory settings. The sequence of the entire genome of *T. castaneum* has been publicly available since 2008^1^, and transposable element-mediated transgenesis of this insect has been successful^2^. Most importantly, this beetle is highly susceptible to systemic RNA interference (RNAi). Injection of double stranded RNA (dsRNA) into the body cavity at any intended stage successfully suppresses the expression of the target gene in the entire body^3^,^4^. In addition, parental RNAi, which can silence gene expression in developing embryos, has also been a useful tool for studying embryonic development^5^.

Two cell lines for *T. castaneum* have recently been independently developed. One is an adherent cell line that was derived from the late pupal stage^6^ and named BCIRL-TcA-CLG1 (TcA). Another embryonic cell line that forms vesicles in suspended culture, named Tc81, was subsequently developed specifically to study juvenile hormone responses^7^. These are the first immortalized cell lines derived from *T. castaneum* and represent major additions to the *Tribolium* tool kit.

Here, we have enhanced the utility of the TcA cell line by characterizing its patterns of gene expression using next-generation sequencing techniques. Further, we have also demonstrated the extreme sensitivity of TcA gene expression to dsRNA silencing and identified and validated two TcA-specific promoters, one constitutive and one inducible, that produce significant levels of transgene expression. In combination with the genomic tools available for *Tribolium*, these enhancements make the TcA cell line an invaluable tool for exploring basic molecular and cellular physiology of the red flour beetle.

## Results and Discussion

### Transcriptome

**Figure 1:**
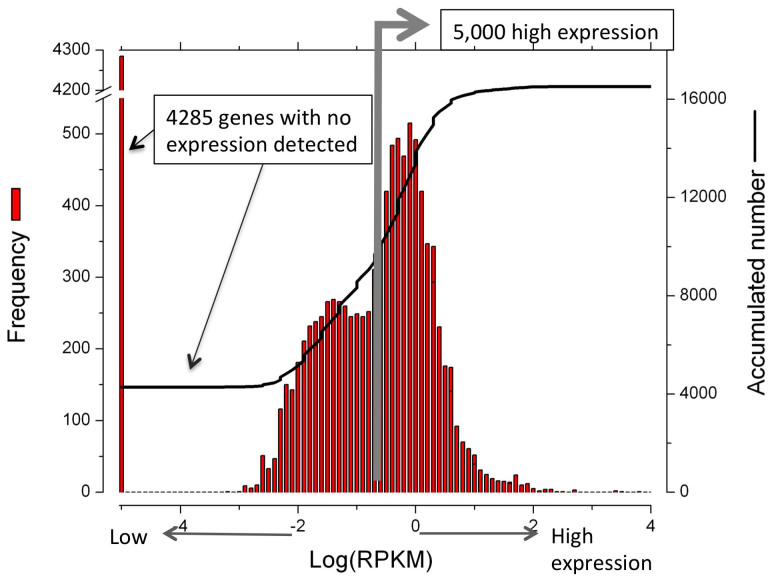
Frequency distribution of TcA transcriptome data compared to the Official Gene Set (OGS) of *Tribolium castaneum* Tcas 3.0. 4,285 genes were not detected (Down0) and 5,000 genes had relatively high expression levels (Up5k).

Illumina sequencing generated 144 million raw single reads (50 bp each). About 105 million reads were mapped to the Tcas 3.0 genome sequence covering 12,265 genes (74%) of the 16,550 in the *T. castaneum* official gene set. We performed gene ontology-term (GO-term) enrichment analysis for the 5,000 most highly expressed genes (Up5k) and the 4,285 undetected genes (Down0), as ranked by RPKM – reads per kilobase in a million reads (Fig. 1).

**Figure 2:**
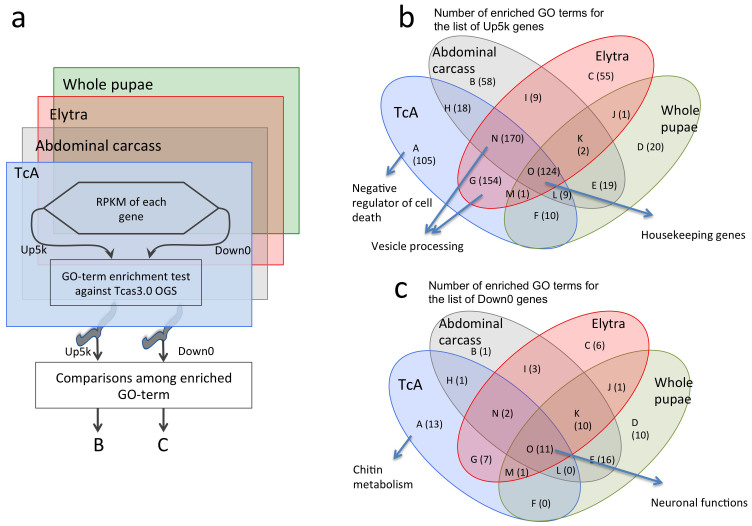
Enrichment tests for the TcA transcriptome. (a) Schematic diagram explaining the approach to perform the enrichment test comparing the transcriptome data of TcA to RNAseq data from abdominal carcass, elytra, and whole pupae. Enriched gene ontology (GO) terms for the top 5,000 genes and the genes with no transcript of each library were compared. (b) Venn diagram showing the numbers of common GO terms found for Up5k in each library with the representative categories of GO terms (c) Venn diagram showing the numbers of common GO terms found for Down0 in each library with the representative categories of GO terms.

Enriched GO-terms from the TcA Up5K and Down0 groups were compared with those from abdominal carcass, elytra, and whole pupae transcriptome sequencing projects (Fig. 2a). A total of 591 GO-terms were found to be significantly enriched in the TcA Up5k (RPKM>0.4686) group; 124 of which were found in the Up5k group of all four RNAseq databases (O in Fig. 2b). The enriched GO-terms for both the Up5k and Down0 had the highest similarity to that of the elytra RNAseq data, with the abdominal carcass and whole pupae comprising the second and third similar collection of enriched GO-terms. The similarity of the TcA enriched GO-terms with those of the elytra suggest that the TcA cell line may be of an epidermal origin (Fig. 2a, b, c).

The enriched GO-terms commonly found in all four RNAseq for the Up5k (O in Fig. 2b) generally included housekeeping genes, including those for ribosomal proteins (GO:0005840) and subunits (GO:0015934 and 0015935), as well as mitochondrial respiratory components (GO:0005739, 0005740, 0005743, 0005746, 0005747, 0005753, and 0005759). The commonly enriched GO-terms in Down0 groups of all four RNAseq datasets included genes involved in sensory neural function (Fig. 2c, GO:0007600, 000718, 0007606, 0038023, 0004930, 0004888). Large numbers of enriched GO-terms for Up5k were shared between TcA, abdominal carcass, and elytra (N in Fig. 2b) or between TcA and elytra (G), while the similarity was relatively low between TcA and whole pupae (F). Among the GO-terms in the category N (common to TcA, abdominal carcass, and elytra in Fig. 2b), it is noteworthy to mention that many of the GO-terms are related to intracellular vesicle formation and activity (GO:0006897, 0006909, 0006911, and 0016192), as it was also the case with category G (common in TcA and elytra; GO:0030133, 0006888, and 0048193). In category A (uniquely enriched only in TcA), it is interesting to note that GO-terms related to negative regulators of cell death (GO:0043066 and 0043069) are among those in the Up5K group and may be partly responsible for the establishment of the immortalized, stable cell line.

**Figure 3:**
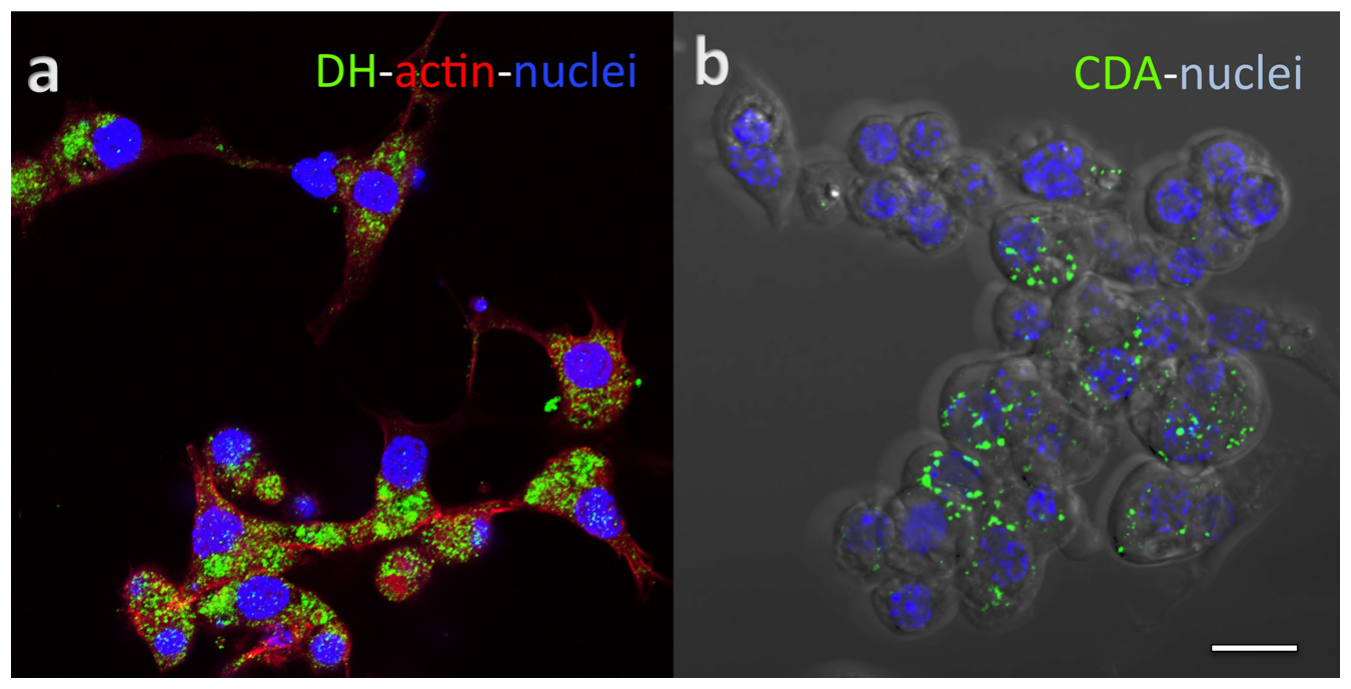
Immunocytochemistry of TcA showing positive reactions to anti-diuretic hormone antibody and anti-chitin deacetylase antibody. (a) immunoreactivity for calcitonin-like diuretic hormone (green), actin (phalloidin, red), and nuclei (DAPI, blue) are shown. (b) immunoreactivity for chitin deactylase (green) and nuclei (DAPI, blue). Scale bar is 10 μm.

We used immunohistochemistry to confirm the presence of proteins from genes that were highly expressed in the tanscriptome. An antibody specific for a signaling peptide, calcitonin-like diuretic hormone^8^, showed small cytoplasmic vesicles in every cell (Fig 3a), indicating a uniformity of expression among the TcA cells. However, an antibody specific for chitin deacetylase stained vesicles in only a subpopulation of TcA cells (Fig. 3b), suggesting some variability in gene expression between cells.

### Expression of the genes involved in cuticle and chitin metabolism

Our transcriptome data showed that the TcA cell line highly expresses a set of genes associated with chitin metabolism and or cuticle structure (Table S2). We also found a subset of cuticle proteins (Table S1), including CP6 (TC013135, RPKM 41.58) and ld-cp1v1 (TC000442, 7.537), that are highly expressed. In addition, chitinase 20, 21, and 11N were expressed at moderate levels. Other genes that are known to have critical functions in proper cuticle formation that were included in the Up5k group of TcA were Rtv (TC007364, RPKM 38.16), Obstructor A1 (TC011140, 10.91), tyrosine hydroxylase (TC002496, 6.665), laccase 1 (TC000821, 6.103), chitin deacetylase 1 (TC14100, 2.589), and KNK1 (TC010653, 2.219). Most notable is low, but significant levels of expressions of both chitin synthase 1 (42 reads, RPKM 0.06) and 2 (67 reads, 0.11) genes.

Although a number of genes involved in cuticle metabolism were found to be highly expressed, supporting the idea of TcA as an epidermal cell type, there were a large number of chitin metabolic genes that were captured in Down0. Indeed, category A, enriched only in TcA with no detectable expression (Down0. Fig. 2c), included many GO-terms for chitin catabolism and chitinase activities. Therefore, it seems TcA expresses a specific subset of cuticle proteins and chitinases at significant levels while it lacks expression of many other genes involved in cuticle metabolism, perhaps because the TcA cell line is a clonal descendent from a specific subtype of epidermal cells. It would be of interest in the future to determine if the TcA line can be used as a model as an insect epidermal cell type with the activity for chitin synthesis.

In addition to the genes involved in chitin metabolism discussed above, we also found that numerous genes putatively involved in insect immunity^9^ are expressed in the TcA cell line. These are listed in Table S3 and entire expression data is in data set 1.

### Expression of genes encoding candidate RNAi machinery

RNAi is very effective in *T. castaneum* during all life stages, and has been widely used to study candidate gene function^10^,^11^. Previous studies have established the basic components of the RNAi pathway that are found in the *T. castaneum* genome^4^,^12^. Based on the information from these studies, we examined the expression of genes encoding core RNAi machinery (Table 1) and genes implicated in systemic RNAi (Table 2) in the TcA cell line. All genes encoding components of the cellular RNAi machinery were expressed in the TcA cell line (Table 1) including the three dicer family genes (dicer 1, dicer 2, and drosha), the genes for their associated dsRNA binding proteins (loquacious, R2D2 and C3PO, and pasha), all 5 argonaute genes (Ago 1, 2A, 2B, 3, and piwi), and the exonuclease, snipper. In addition, many of the candidate genes thought to be associated with the systemic RNAi response are expressed in the TcA cell line (Table 2). The apparent intact nature of the RNAi response system in the TcA cell line vastly increases the potential for this cell line to be used in highly informative investigations of cellular and molecular physiology.

### TcA-specific promoters for reporter gene expressions

Heterologous expression of a native, chimeric, or fluorescently-tagged protein in a cell line is a commonly used and extraordinarily useful tool. Use of the TcA for expression of target proteins depends on efficient promoters that constitutively or conditionally promote the expression of the exogenous gene. Our initial trials with promoters that are commonly used in insect cell lines were not successful at expressing either the fluorescent reporter protein, Tomato, or enhanced green fluorescent protein, EGFP. In a test of the common promoters frequently used in insect cell systems, the Actin 5 promoter of *D. melanogaster*, the CMV promoter in the pcDNA3.1+ plasmid, the OpIE1 and OpIE2 promoters in the pIZT vector (Invitrogen), and the alpha tubulin promoter of *T. castaneum*^13^ were all ineffective at producing detectable levels of Tomato up to 3 days after transfection of the plasmid into TcA cells.

Therefore, we expanded our efforts to test endogenous *Tribolium* promoters. We chose candidate promoters based on the expression levels of genes in the TcA (~>5 in the RPKM), defined small sizes of intergenic regions at the 5′ end of the genes, and a lack of large introns. The putative promoters for three genes encoding ribosomal protein subunits, TC014362, TC000476, and TC006550, and one heat shock protein 68 (hereafter named as TcHS70 by *Drosophila* homology) were identified and selected for testing. Two promoters, TC006550 encoding a 40S ribosomal protein subunit and TcHS70, were found to be active for production of the reporter protein.

**Figure 4:**
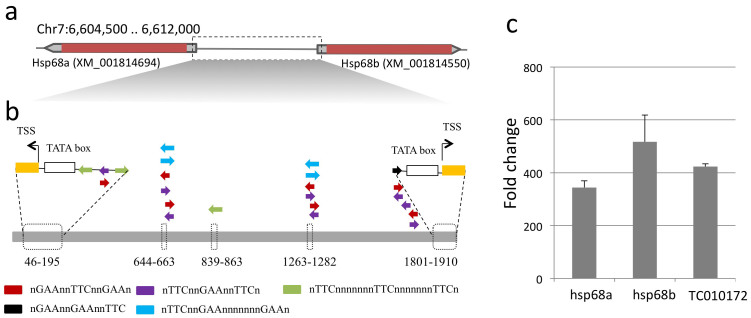
TcHS70 promoter structure and TcA response to heat shock for increased transcripts. (a) Genomic structure of the heat shock proteins 68a and 68b. (b) The promoter region containing the heat shock factor binding motifs, putative TATA boxes and transcription start sites. (c) Induction of expression of heat shock proteins 68a, b, and another homologous sequence TC10172 in TcA, determined by quantitative RT-PCR with rps3 normalization.

**Figure 5:**
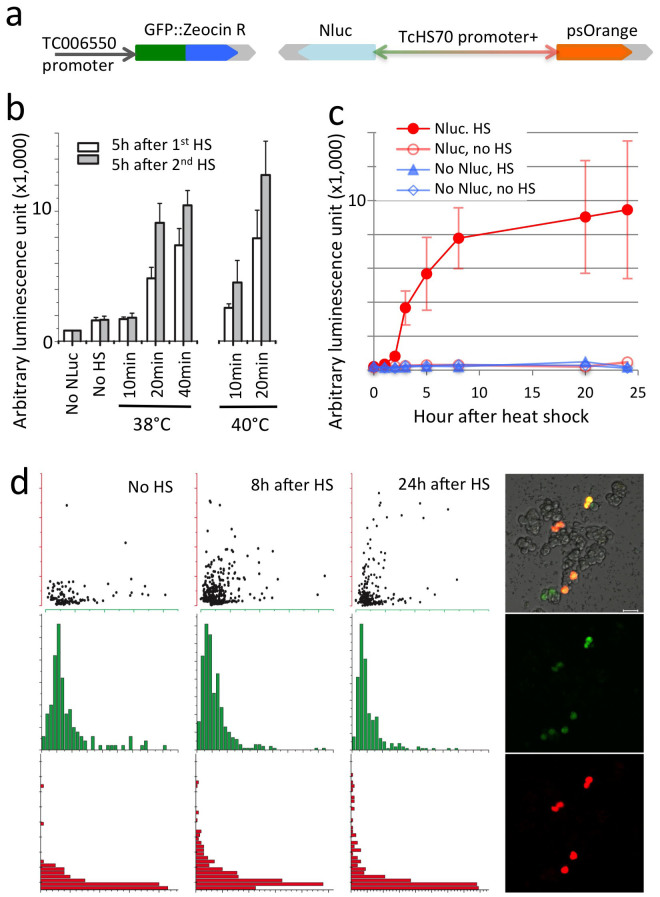
Heterologous expression of the reporter genes GFP::Zeocin R, nanoluciferase (Nluc) and photo-switchable Orange fluorescent protein. (a) Construction of the heterologous expression plasmid using the pUC18 backbone with ampicillin resistance for expression of the reporter genes by the TC006550 (rpS40 protein) and TcHS70 promoters. (b) Induction of Nluc expression by different temperatures, durations, and numbers of heat shock. (c) Timecourse of Nluc activity in the media following heat shock (38°C for 30 min). (d) Green fluorescence from EGFP expression (green) and orange (red) fluorescence from psOrange over time after heat shock. The histogram was made by sampling more than 300 cells for each time point. The right panel shows an example of a confocal image for cells expressing both green and red fluorescence. Note that two cells on the bottom left of each photo are green, but not red, showing auto fluorescence of large vacuolar structures in those TcA cells.

The TcHS70 contained multiple putative heat shock sequence elements (Fig. 3a and b). Initially, we tested the TcHS70 activity after a heat shock in the endogenous cell. Immediately after a heat shock of TcA cells at 40°C for 30 min, total RNA was extracted to investigate the transcriptional responses of the hsp genes. We found ~300 to 500-fold increases in mRNA levels for each hsp68a, hsp68b, and another highly similar hsp TC010172 after normalization to rps3 transcript levels and comparison to the no heat shock control (Fig. 4c). We subsequently used the 1910bp intergenic region between the coding genes for hsp68a and hsp68b as the TcHS70 promoter (Fig. 4a and 5a). This sequence included the putative TATA boxes, transcription start sites located in each end, and numerous heat shock sequence elements (Fig. 4b).

We constructed a plasmid using the pUC18 vector as a backbone, containing reporter and antibiotic proteins: the TC006550 promoter driving expression of an EGFP-Zeocin resistance fusion protein and the bidirectional TcHS70 promoter driving expression of secretory nanoluciferase (Promega) and photo switchable Orange^14^ (Fig. 5a). Transient transfection of this construct was followed by heat shocks at 38°C for 10, 20, and 40 min. and at 40°C for 10 or 20 min. The naouluciferase activity in the cell culture media was significantly increased 5 hr after heat shock except in the case of 38°C for 10 min. Application of a second heat shock further increased naoluciferase activity compared to the first heatshock (Fig. 5b). The changes in nanoluciferase activity over time indicated that the minimum expression and maturation time for nanoluciferase activity is ~3 hr after heatshock, and it reaches a plateau at ~8 hr following heat shock at 38°C for 40 min (Fig. 5b and c). The cells with constitutive expression of EGFP were also found to have inducible expression of psOrange following heat shock, although induction of visible levels of psOrange appears to require a longer time for maturation than that of nanoluciferase. Occasionally, we found autofluorescence in the TcA that is similar to the EGFP wavelength. This autofluorescence was limited to large vacuolar structures appearing only in a small subpopulation of the cells (Fig. 5d).

In conclusion, based on expression of the reporter proteins, the TcHS70 is a highly inducible bidirectional promoter. Further, the TC006550 promoter also provides a moderate level of constitutive reporter expression. The plasmid containing those promoters will be a valuable tool for expression of heterologous genes.

### RNAi in the TcA cell line

**Figure 6:**
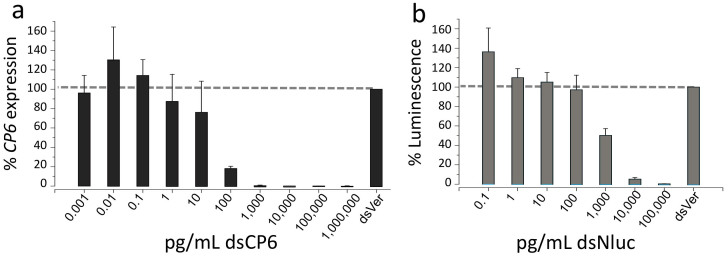
RNA interference of the cuticle protein CP6 and nanoluciferase. (a) Effects of different concentrations of dsRNA in the culture media on mRNA expression of CP6 normalized to that of rps3. (b) Efficiency of RNAi in TcA cells transiently transfected with the Nluc expression plasmid. Bars in (b) indicate % luciferase activities of the nanoluciferase.

Expression of many of the components of the RNAi machinery led us to investigate the sensitivity of this cell line to RNAi. Accordingly, we selected a relatively highly expressed chitin protein, CP6, as our target for silencing, and used the common control target, vermilion, as a negative control. dsRNA was synthesized (231 bp for CP6, 400 bp for vermilion) and mixed directly with culture media at various concentrations (1 fg/mL to 1 µg/mL, vermilion was mixed at 100 ng/mL) prior to applying to the TcA cells. qPCR quantification showed that the TcA cell line is highly sensitive to RNAi of CP6. Treatment with as little as 1 ng/mL resulted in >99% suppression of CP6 mRNA expression (Fig. 6a). Suppression of CP6 mRNA decreased in a dose-dependent manner as dsRNA concentration decreased below 1 ng/mL, with no significant reduction in CP6 expression occurring with 1 pg/mL dsRNA. The sensitivity to dsRNA of TcA cells is similar to that of the Tc81 cell line as measured with dsRNA targeting the *met* gene^7^, and more than 10,000 times greater than that for the S2 *Drosophila* cell line^15^.

We also tested our CP6 dsRNA *in vivo* by injecting dsRNA (2 or 200 ng/beetle) into *T. castaneum* early instar larvae to ascertain the effects of CP6 RNAi *in vivo*. No obvious phenotype was associated with silencing of CP6 in any developmental stage (late instar larvae, pupae, or adult), although the beetles injected with vermilion dsRNA showed the characteristic white eyes associated with this treatment. Analysis of CP6 mRNA showed that both 2 and 200 ng treatments per individual resulted in >99% suppression of expression at the organismal levels without noticeable phenotype in the observation until the early adult stage.

We also subsequently tested the efficiency of RNAi to silence the expression of heterologously expressed proteins, transiently transfected for heat shock inducible nanoluciferase (Fig. 6b). Silencing of nanoluciferase activity induced by heat shock was measured following treatment with dsNluc. Interestingly, significant suppression of nanoluciferase activity required a minimum of 1 ng/mL (~50% suppression, Fig. 6b), which is more than 10-fold greater than that required for suppression of CP6 expression. We suspect that this difference is likely caused by the rapid induction of extremely high levels of expression of the nanoluciferae following heat shock treatment.

High sensitivity of the TcA cells to dsRNA in the media requires efficient mechanisms for cellular uptake of primary dsRNA followed by dsRNA processing into small interference RNA (siRNA) which forms complexes with the target messenger RNA. Our results show that TcA expresses a large number of candidate molecular components of the RNAi pathway (Table 1) and shows high sensitivity to dsRNA, provideing an excellent opportunity to explore novel, highly efficient RNAi mechanisms in the TcA cell.

## Methods

### Cell culture

TcA, an adherent cell line, was derived from BCIRL-TcA-CLG1 that was originally established from the late pupal *T. castaneum*^6^. TcA cells were grown in ExCell 420 media (Sigma, St. Lois, MO.) supplemented with 10% insect cell culture-tested fetal bovine serum (Atlanta Biologicals, Lawrenceville, GA) and 4.5 µg/ml of bovine insulin at 27°C and 60% relative humidity, although bovine insulin was found to be not necessary for normal cell growth in the later study (Park et al, unpublished data).

### Transcriptome sequencing and analyses

Total RNA was harvested from confluent cells using a commercial kit (Qiagen, Valencia, CA.). RNA quality was tested using an Agilent Bioanalyzer prior to submission for sequencing. Illumina-based (GXII platform) rRNA-depleted transcriptome sequencing of a single RNA sample was carried out by Cofactor Genomics (St. Louis, MO.). Obtained sequences were single direction, 50 nt in the length, and filtered for the sequences with a q-score higher than 30 and longer than 30 nt by using the FastX toolkit implemented in DNA Galaxy^16^. Assembly of the sequences to the reference genome Tcas3.0 was made in DNA Nexus (https://classic.dnanexus.com/home). The RPKM (read per kilobase per million mapped reads) was used for identifying the top 5,000 highly expressed genes (Up5k) and the genes with undetectable transcript levels (Down0) (Fig. 1).

The transcriptome of TcA was evaluated by comparisons of enriched GO-terms to other tissue specific RNAseq data. Direct comparisons of RNAseq data for expression levels of each gene by RPKM in different sets of data were not possible because of varying sample preparation procedures and sequencing approaches in each RNA library. For example, normalization of our sequence data to another library introduced largely skewed raw RPKM values, and was thus not appropriate to use in conventional methods for comparing expression levels. As an alternative approach, the lists of genes for the Up5k and Down0 were used for a GO-term enrichment test against the official gene set (Tcas 3.0 OGS^17^,) in Blast2GO^18^ at the False Discovery Rate (FDR) of 0.05 (Fig. 2). Enriched GO-terms of each library representing RNAseq data from elytra of late pupal stage (elytra, the data provided by Dr. S. Muthukrishnan), abdominal carcass excluding the gut and the central nervous system in the early adult stage (abdominal carcass, data provided by Dr. T. Phillips), and whole pupal stage (whole pupae, data provided by Dr. G. Butcher) were compared for each Up5k and Down0.

### Immunohistochemistry

Anti-DH37 (rabbit, 1:500), originally raised against the diuretic hormone 37 of *Tenebrio molitor*^8^, and anti-CDA1 (rabbit, 1:500) against *T. castaneum* chitin deacetylase 1^19^ were used to label their respective targets in fixed TcA cells grown on a petri dish. A goat anti–rabbit IgG antibody conjugated with Alexa Fluor 488 (Life technologies) was used as the secondary antibody. Additionally, we used phalloidin conjugated with Alexa Fluor 594 (Life technologies) to stain actin and 4′,6′-diamino-2-phenylindole (DAPI 300 nM, Sigma) to stain cell nuclei (Fig. 3). Images were captured on an LSM 700 confocal microscope under a 40X objective (Zeiss).

### Plasmid construct

pIZTV5-His (Invitrogen), the plasmid originally developed for lepidopteran insects, was used for constructions of other variants. Plasmids we constructed were for testing putative promoters in the TcA cell line for expression of fluorescent protein dTomato^20^ as the reporter. The promoters tested were: the CMV promoter from the pcDNA3.1+ plasmid (Invitrogen), the OpIE1 and OpIE2 promoters from the pIZT vector, the *Tribolium* alpha tubulin promoter^13^, and the *Tribolium* heat shock promoter (TcHS70, in this study). The dTomato was amplified using the primers dTomatoF and dTomatoR (Table S1) and cloned into a pGEMT easy vector. Then, dTomato was introduced into the pIZTV5-His vector by using a NotI restriction site (pIZT-dTomato). Subsequently, the promoters were cloned into the pGEMT easy vector and then inserted into pIZT-dTomato replacing the OpIE2 promoter by digestion with the corresponding restriction enzymes and ligation (Figure S1). Among these constructs, the plasmid containing the *Tribolium* heat shock promoter was further modified to add an ampicillin selection marker driven by a *bla* promoter. The OpIE1 promoter upstream of the GFP-Zeocin chimeric gene in pIZTV5-His was replaced by the putative promoters for three genes encoding ribosomal protein subunits, TC014362, TC000476, or TC006550. Subsequently, the constitutively active promoter TC006550 (upstream ~1.2 kb) was used for the expression of GFP-Zeocin.

After we tested promoter efficiencies by using the plasmids described above, we constructed the plasmid that contains several reporter genes in Figs. 4, 5, and S1. The plasmid contains a *bla* promoter with ampicillin resistance, a TC006550 promoter for GFP::Zeocin resistance (Invitrogen) with SV40 3′ UTR, a TcHSP70 bidirectional promoter for secretory nanoluciferase (from pGL3 of Promega) with a BGH 3′UTR, and psOrange^14^ with OpIE2 3′UTR (Invitrogen). The primers used in the construction of the plasmid are listed in Table S1 and the plasmid map including the major restriction sites that were used for plasmid construction is shown in Figure S1.

PCR was conducted using the high fidelity DNA polymerase PrimeStar (Takara). All the restriction enzymes, the T4 DNA ligase as well as the competent cells, DH5α™ high efficiency, were purchased from New England Biolabs.

### RNA interference

dsRNA was synthesized using the Ampliscribe T7 Flash Transcription Kit (Epicentre Biotechnologies, Madison, WI) from cDNA templates using gene-specific primers (Table S1) with a T7 nucleotide sequence attached to the 5′ end of the primer sequences of *CP6* (cuticle protein 6, or also named as CPR71), vermilion, and nanoluciferase. The dsRNA was purified using a commercial kit (MEGAClear, Life Technologies) and run on a 2% agarose gel to confirm the band size. dsRNA was quantified using a Nanodrop Spectrophotometer (Thermo Scientific, Wilmington, DE) and diluted to the desired concentration with nuclease-free water.

For *CP6* RNAi experiments in cell culture, TcA cells were seeded onto 24-well plates (5 × 10^6^ cells/mL) and left to grow for at least 24 h. RNAi was achieved in TcA cells by adding dsRNA for *CP6* or vermilion directly to the culture media. RNA was harvested after 96 h using TRIzol reagent (Life Technologies), and cDNA was synthesized using the ImProm-II™ Reverse Transcription System (Promega). cDNA samples were then diluted 20-fold and *CP6* expression was quantified by qPCR utilizing comparative Ct^21^ on a Biorad thermal cycler (Biorad) with ribosomal protein S3 (rpS3) serving as the internal loading control. Gene-specific primers for CP6 and rpS3 are listed in Table S1.The thermal cycling conditions were as follows: 95°C for 2 min, 40 cycles of 95°C for 15 sec, 60°C for 15 sec and 72°C for 30 sec. Melting curve analysis (55–95°C) and gel electrophoresis were applied to all reactions to ensure consistency, specificity, and amplicon size of the amplified product. All *CP6* Ct values were first normalized to rpS3 in the same sample, and then secondarily to the vermilion treatment in order to produce a relative expression value. These experiments were replicated three times and the results are presented as the mean ± standard error.

For *in vivo* RNAi experiments of *CP6*, early last instar larvae were microinjected with dsCP6 (200 ng or 2 ng/individual) or vermilion (200 ng/individual) dsRNAs. Two weeks post-injection, adults were visually examined for any defects in development. Four individuals from each treatment group were pooled for total RNA isolation. cDNA was synthesized from total RNA (1 µg/rxn) using the ImProm-II™ Reverse Transcription System (Promega). RNAi efficiency was determined by qPCR as described above.

For RNAi of nanoluciferase, TcA cells were seeded onto a 96-well plate (5 × 10^6^ cells/mL) and left to grow for at least 24 h. The TcA cells were transfected with the expression plasmid (Fig. 5a), using FuGENE HD transfection reagent (Promega). Subsequently, various concentrations of dsRNA for nanoluciferase were directly added to the culture media for final dsRNA concentrations ranging from 100 fg/mL to 100 ng/mL. About 48 h after the transfection, the cells were subject to heat shock (HS) at 38°C for 40 min. Approximately 18 h after the initial HS, the media was changed to new media and one more HS was made at 38°C for 40 min. Six hours after the HS, secretory nanoluciferase activity in the cell culture media was measured using the Nano-Glo luciferase assay system (Promega). dsVermilion (100 ng/mL) was used as a negative control.

## Author Contributions

K.S., H.J., J.F., Y.P. performed the experiments. K.S., H.J., J.F., T.W.P., Y.P. provided and analyzed the data. K.S., Y.P., H.J., J.F. and R.W.B. wrote the paper. Y.P. and R.W.B. supervised the project.

## Supplementary Material

Supplementary Information Supplementary data

Supplementary Information Dataset

## Figures and Tables

**Table 1:** Expression of genes encoding core RNAi Machinery in the TcA cell line

Description	Name	TC #	RPKM	Raw Reads
Dicer Family	Dicer 1	TC001750	0.2305	183
	Dicer 2	TC001108	3.455	2426
	Drosha	TC016208	0.8303	382
dsRNA Binding	R2D2	TC008716	0.9222	128
	C3P0	TC007013	0.02626	46
	loquacious	TC011666	0.4626	77
	pasha	TC015332	0.3076	75
Argonautes	Ago 1 (miRNA)	TC005857	4.813	2197
	Ago 2a (siRNA)	TC011525	0.09176	34
	Ago 2b (siRNA)	TC013762	7.539	2950
	Piwi	TC008711	8.248	3174
	Ago 3	TC008511	0.8715	337
Exonuclease	Snipper	TC001174	0.3474	35

**Table 2:** Expression of genes associated with systemic RNAi in the TcA cell line

Description	Name	TC#	RPKM	Raw Reads
Systemic RNAi – dsRNA uptake	SilA	TC011760	0	0
	SilB	TC006161	0.04313	14
	SilC	TC015033	0.6015	200
Systemic RNAi – dsRNA export	Epsin-like (rsd-3)	TC012168	1.877	439
	liquid facets (Epn-1)	TC005393	1.174	261
RNA-dependent RNA polymerase	Elp-1 (RNA-dep RNA pol)	TC015781	0.6645	337
Eater	Odoerant Receptor 138	TC002053	0	0
	Eater XP_969372*	TC030481	0.002727	1
		TC015258	0	0
Endosome Transport	Arf72A	TC008443	0.5325	38
	Rab 7	TC006036	3.107	219
Endocytosis	AP 50	TC011923	6.401	1215
	Clathrin hc	TC015014	7.213	5255
Exocytosis	IdICP	TC010886	0.5782	140
Lysosomal Transport	Light	TC015204	0.8023	290
Rhodopsin-mediated signaling	Nina C	TC014087	0.002047	1
ATP Synthase/ATPase	Vha16	TC011025	10.36	717
	VhaSFD	TC006281	5.631	1288
Metabolism	Gmer	TC014956	0.9353	129
Lipid Metabolism	P13K59F	TC000620	0.6661	269
	Saposin r	TC000449	16.11	5872
Innate Immune Response/phagocytosis	Odoerant Receptor 138	TC002053	0	0
	Eater XP_969372*	TC030481	0.002727	1
	Sr-CI CG4099	TC015258	0	0
	Sr-CII	TC015640	0.08078	19
Oogenesis	Egghead	TC008154	0.4128	83
Translation Regulation	LOC662027 similar to CG5434-PA (Signal recognition particle protein 72)	TC012172	1.008	285
GDP-fucose transmembrane transporter	Gfr CG9620	TC012326	0.1036	15
GDP-mannose 4,6-dehydratase	Gmd CG8890	TC004005	0.1879	29
Peptidase	Unknown	TC002692	0.3452	70
Signal Transduction	Unknown	TC007768	0.2769	51
Ubiquitin Ligase	Unknown	TC004152	1.746	3029
Peptidase	Unknown	TC016254	0.2672	105
Zinc Finger Transcription Factor	Unknown	TC009067	0.6178	78
Unknown		TC012410	0.3755	138
		TC014009	0.6331	49
	LOC658010 similar to kinase D-interacting substance 220	TC004825	0.3245	86
	LOC655368 similar to CG31974-PA	TC007973	4.659	282
