# Supplementary material for: The Tribolium castaneum cell line TcA: a new tool kit for cell biology
Source: Sci Rep. 2014 Oct 30;4:6840. doi: 10.1038/srep06840 (PMC4213800; doi:10.1038/srep06840)

**Supplementary material**

**The *Tribolium castaneum* cell line TcA: a new tool kit for cell biology**

Kristopher Silver^1,*^, Hongbo Jiang^2,*^, Junping Fu^2^, Thomas W. Phillips^2^, Richard W. Beeman^2,3^, and Yoonseong Park^2^.

^1^Department of Anatomy and Physiology, Kansas State University, Manhattan, KS 66506.

^2^Department of Entomology, Kansas State University, Manhattan, KS 66506.

^3^USDA, Agricultural Research Service, Center for Grain and Animal Health Research, 1515 College Ave, Manhattan, KS 66502.

*K. Silver and H. Jiang contributed equally to this work.

Table S1. Primers used in this study

Table S2: Expression of Cuticle- and Chitin synthesis-associated genes in the TcA cell line.

Table S3: Expression of immunity-related genes in the TcA cell line.

Figure S1.

The map for the plasmid used in this study. The sequence is deposited in NCBI with the GenBank Accession ############.

Table S1. Primers used in this study

| Experiments | Targets | Primer information |  |
| --- | --- | --- | --- |
|  |  | Forward | Reverse |
| PCR and cloning | pIZT backbone | pIZT-F1  *CGAATTTAAAGCTTGGTACCG | pIZT-R1  **GG*GGTACC*CC*GCATGCCCAGACATGATAAGATACATTG |
|  |  | pIZT-F3  *GGA*AGATCT*TCC*GGTGAGGAACTAAACCATGG | pIZT-R5  *TT*GGCGCGCC*AA*GGAAGATCTTCCGGTCTGAC |
|  | bla-Amp | blaPro  *TT*GGCGCGCC*AA*TGCGCGGAACCCCTATTTG | blaAmp-R  *GGA*AGATCT*TCC*GGTCTGACAGTTACCAATGC |
|  | dTomato | dTomatoF  ATGGTGAGCAAGGGCGAG | dTomatoR  TTACTTGTACAGCTCGTCCATG |
|  | TcHS promoter | KpnI-HSP-F  *GG*GGTACC*CC*GCTTGCTTAGCTTTGTTGC | KpnI-HSP-R  *GG*GGTACC*CC*TCAAATTCACTGACAGCGC |
|  | Tc006550 Promoter | 6550ProF  *TT*GGCGCGCC*AA*CAGCCGTTTTATTGCTTTCC | 6550ProR  *TT*GGCGCGCC*AA*CCTTACGTTAGAATTGAGTTACG |
|  | Tc014362 promoter | 14362ProF  *TT*GGCGCGCC*AA*GGGTGCTGAACAACTTAAGTC | 14362ProF  *TT*GGCGCGCC*AA*TTTTCACCTTTGAGACACACA |
|  | Tc000476 promoter | 476ProF  *TT*GGCGCGCC*AA*GCCCTAAATGACAAACGC | 476ProR  *TT*GGCGCGCC*AA*TTTGAACAAGGTAGATGTGAAC |
|  | Tcα-tubulin promoter | α-tubF1  *TT*GGCGCGCC*AA*TACCGACATGGCGGGGAG | α-tubR1  *TT*GGCGCGCC*AA*ACCCCGATTGTTTAGCTTGT |
|  | CMV promoter | CMVpro-F-KpnI  *GG*GGTACC*CC*GCGTTGACATTGATTATTGAC | CMVpro-R-KpnI  *GG*GGTACC*CC*GAGAGCTCTGCTTATATAGACC |
|  |  |  |  |
| dsRNA synthesis | TcVermillion | dsTcVer-F  taatacgactcactatagggGAGCAAATCGCCAAGTCGG | dsTcVer-R  taatacgactcactatagggCCTGGGTTCGTCCCTGTAA |
|  | TcCP6 | dsCP6-F  taatacgactcactatagggCGCCTTGCCATGGACTGGACC | dsCP6-R  taatacgactcactatagggCGCATCCCCGTTTTCCGGGT |
|  | Nanoluciferase | dsNlucF  taatacgactcactataGGGAGGTGTGTCCAGTTTG | dsNlucR  taatacgactcactataggGTTGATCAGGCGCTCGTC |
|  |  |  |  |
| qPCR | HSP68a | 68-qF  ATACGAAGATAGACAGAAACAGC | 68a-qR  ACTAAGTAGGAACAAAAACCGA |
|  | HSP68b | 68-qF  ATACGAAGATAGACAGAAACAGC | 68b-qR  CATATACAACAAAAGGATTTAAC |
|  | Tc010172 | 10172-qF  GTACCAACAAGGCGGTCAG | 10172-qR  CAATAGTGACTGCATTTAACATAATAC |

Star marks (*) are for phosphorylations at the 5’ end of the primers. Underlined nucleotides are the restriction sites introduced in the primers. Italic nucleotides are additional bases attached to end of the DNA molecule to ensure the effective cleavage close to the hangout of the PCR products. Nucleotides in lowercases are the T7 promoter used for the dsRNA synthesis.

Table S2: Expression of Cuticle- and Chitin synthesis-associated genes in the TcA cell line.

| Description | TC# | RPKM | Raw Reads |
| --- | --- | --- | --- |
| cuticle protein cp6 (TcCPR71) | TC013135 | 41.58 | 2553 |
| ribosomal protein s2 (TcRtv) | TC007364 | 38.16 | 4603 |
| protein yellow (Tcyellow-1) | TC005444 | 22.04 | 4518 |
| imaginal disc growth factor 4 | TC013917 | 13.58 | 2536 |
| cuticular protein analogous to peritrophins 3-a1 (TcObst-A1) | TC011140 | 10.91 | 1123 |
| yellow protein (Tcyellow-F) | TC005565 | 8.636 | 1699 |
| cuticular protein ld-cp1v1 | TC000442 | 7.537 | 642 |
| tyrosine hydroxylase | TC002496 | 6.665 | 1588 |
| imaginal disc growth factor 4 | TC013918 | 6.192 | 1178 |
| laccase 1 | TC000821 | 6.103 | 1842 |
| integrin beta subunit (agap000815-pa) TcPSBI-1 | TC011707 | 4.818 | 1756 |
| dopamine n acetyltransferase | TC008204 | 4.806 | 534 |
| chitin deacetylase 4 | TC007635 | 4.428 | 940 |
| yellow-b | TC005480 | 4.383 | 796 |
| brain chitinase and chia (chitinase20) | TC009872 | 3.904 | 758 |
| cuticular protein analogous to peritrophins 3-b (TCObst-B) | TC011139 | 3.461 | 419 |
| yellow-c | TC016299 | 3.273 | 576 |
| cuticular protein analogous to peritrophins 1-c | TC000316 | 3.003 | 557 |
| cuticular protein rr-1 family (agap000344-pa) | TC001118 | 2.783 | 290 |
| cuticular protein rr-1 family (agap002726-pa) | TC015304 | 2.619 | 205 |
| chitin deacetylase 1 | TC014100 | 2.589 | 599 |
| cuticular protein analogous to peritrophins 3-e (TcObst-E) | TC011349 | 2.294 | 246 |
| domon domain-containing protein (TcKNK1) | TC010653 | 2.216 | 643 |
| n-acetyltransferase 2 | TC007905 | 2.187 | 208 |
| cuticular protein rr-1 family (agap006497-pa) | TC013013 | 1.937 | 484 |
| cuticular protein rr-3 family (agap006931-pa) | TC012829 | 1.853 | 258 |
| adult cuticle | TC003507 | 1.683 | 123 |
| adult cuticular protein | TC013987 | 1.655 | 136 |
| pupal cuticle protein | TC014771 | 1.376 | 94 |
| multidrug-resistance like protein isoform m (TcSUR) | TC012253 | 1.306 | 1020 |
| syntaxin 1a (TcSyn4a) | TC011694 | 1.094 | 157 |
| cuticular protein | TC009890 | 1.092 | 94 |
| gasp precursor (TcObst1&2) | TC001169 | 1.082 | 291 |
| cuticular protein analogous to peritrophins 3-d2 (TC-Obst-D) | TC001350 | 1.03 | 114 |
| syntaxin 13 (TcSyn 4c?) | TC007177 | 0.9181 | 104 |
| inwardly rectifying potassium isoform b (TcKir1) | TC006706 | 0.8042 | 161 |
| protein yellow (Tcyellow-E or Tcyellow-D) | TC006229 | 0.7772 | 165 |
| syntaxin-like protein (TcSyn1 or 6?) | TC003074 | 0.7709 | 46 |
| larval cuticle protein a3a (tm-a3a) (tm-lcp a3a) | TC000720 | 0.7457 | 59 |
| 10g08 (TcSyn5) | TC007974 | 0.7292 | 169 |
| syntaxin 18 | TC011475 | 0.6586 | 84 |
| chitinase domain-containing protein 1 (chitinase 21) | TC006344 | 0.6005 | 101 |
| domon domain-containing protein (TcSkeletor) | TC010675 | 0.597 | 349 |
| syntaxin 16 | TC009870 | 0.5947 | 72 |
| brain chitinase and chia (chitinase 11N) | TC015665 | 0.5017 | 59 |
| cuticular protein 62bc | TC002434 | 0.4942 | 25 |
| cuticular protein analogous to peritrophins 1-d | TC009263 | 0.481 | 47 |
| cuticular protein rr-2 family (agap006868-pa) | TC007306 | 0.4452 | 36 |
| cuticular protein 62bc cg1919-pa | TC013816 | 0.4336 | 42 |
| syntaxin 8 | TC003137 | 0.4292 | 18 |
| syntaxin 17 | TC003648 | 0.4141 | 53 |
| tan | TC003448 | 0.4134 | 69 |
| multidrug resistance-associated protein 7 (SUR2) | TC008035 | 0.3788 | 246 |
| cuticular protein analogous to peritrophins 1-b | TC000587 | 0.3621 | 31 |
| cuticular protein rr-1 family (agap005995-pa) | TC014501 | 0.361 | 59 |
| isoform d (TcCDA2) | TC014101 | 0.3429 | 86 |
| amp dependent ligase (Tcebony) | TC011976 | 0.325 | 121 |
| cuticular protein analogous to peritrophins 1-a | TC004733 | 0.3093 | 44 |
| udp-n-acteylglucosamine pyrophosphorylase | TC005593 | 0.3065 | 64 |
| cuticular protein analogous to peritrophins 3-a2 (TcObst-A2) | TC011141 | 0.2928 | 30 |
| cuticular protein analogous to peritrophins 3-d1 (TcObst-X) | TC011142 | 0.2828 | 28 |
| pupal cuticle protein 36a | TC013138 | 0.2557 | 22 |
| fused lobes (TcFDL) | TC009779 | 0.2514 | 65 |
| chitin deacetylase-like isoform d (CDA5) | TC006846 | 0.2431 | 117 |
| endocuticle structural glycoprotein bd- | TC013126 | 0.2383 | 17 |
| cuticle protein | TC003356 | 0.2103 | 56 |
| adult cuticular protein | TC013988 | 0.2071 | 12 |
| pupal cuticle protein | TC013136 | 0.1821 | 10 |
| cg1397-pa (TcRtv) | TC007384 | 0.1775 | 35 |
| aromatic amino acid decarboxylase TcDDC3) | TC013402 | 0.1472 | 28 |
| metalloproteinase inhibitor 3 (TcTIMP) | TC002305 | 0.1452 | 13 |
| cuticular protein rr-1 family (agap009876-pa) | TC013139 | 0.1383 | 11 |
| larval cuticle protein a3a (tm-a3a) (tm-lcp a3a) | TC000723 | 0.1362 | 11 |
| pupal cuticle protein | TC013131 | 0.1312 | 8 |
| chitinase 3 (chitinase 16) | TC009176 | 0.1201 | 20 |
| brain chitinase and chia (chitinase 6N) | TC003876 | 0.1191 | 122 |
| yellow | TC000802 | 0.1176 | 21 |
| protein naked cuticle-like protein | TC001637 | 0.115 | 29 |
| pupal cuticle protein | TC013812 | 0.1088 | 8 |
| chitin synthase 2 | TC012163 | 0.1058 | 67 |
| syntaxin 1a (TcSyn4b) | TC012094 | 0.1016 | 13 |
| cuticular protein rr-2 family (agap006867-pa) | TC013815 | 0.09353 | 11 |
| cuticular protein 97ea | TC001119 | 0.08975 | 13 |
| dopa decarboxylase | TC013480 | 0.08746 | 18 |
| tpa: cuticle protein | TC012892 | 0.08695 | 10 |
| cuticular protein rr-2 family (agap001664-pa) | TC010054 | 0.08131 | 9 |
| yellow-h | TC006230 | 0.0789 | 16 |
| major royal jelly protein 4 (Tcyellow-4) | TC002508 | 0.0763 | 13 |
| cuticular protein rr-2 family (agap006868-pa) | TC016307 | 0.07326 | 7 |
| chitin deacetylase 9 | TC003905 | 0.07265 | 12 |
| major royal jelly protein 4 (Tcyellow-5) | TC002509 | 0.07171 | 12 |
| pupal cuticle protein | TC007764 | 0.07116 | 4 |
| cuticle protein cpg42 | TC004827 | 0.06704 | 10 |
| chitin synthase 1 | TC014634 | 0.06004 | 42 |
| cuticular protein analogous to peritrophins 1-h | TC009894 | 0.05894 | 21 |
| cuticular protein 100a | TC001115 | 0.05782 | 5 |
| cuticular protein rr-2 family (agap008960-pa) | TC000719 | 0.05354 | 5 |
| yellow-h cg1629-pa (Tcyellow-3) | TC003898 | 0.04513 | 8 |
| cuticle protein | TC000724 | 0.04303 | 4 |
| cuticular protein rr-1 family (agap006007-pa) | TC013132 | 0.03903 | 4 |
| aromatic amino acid decarboxylase (TcTyrDC1) | TC012567 | 0.03683 | 10 |
| cuticular protein 92f | TC012828 | 0.03544 | 4 |
| laccase-like multicopper oxidase 1 (TcLac2) | TC010490 | 0.03491 | 4 |
| udp-n-acteylglucosamine pyrophosphorylase | TC001751 | 0.03359 | 7 |
| tpa: cuticle protein | TC012893 | 0.03304 | 1 |
| cuticular protein | TC008228 | 0.03257 | 2 |
| cuticular protein rr-1 family (agap009874-pa) | TC013134 | 0.0314 | 3 |
| isoform b (TcKNK3) | TC002304 | 0.03136 | 8 |
| pupal cuticle protein c1b (tm-c1b) (tm-pcp c1b) | TC006262 | 0.03063 | 2 |
| cuticular protein rr-1 family (agap005456-pa) | TC008401 | 0.02877 | 5 |
| dopa decarboxylase (TcDDC2) | TC013401 | 0.02764 | 6 |
| glutamate decarboxylase | TC009324 | 0.0271 | 6 |
| cuticular protein rr-1 family (agap005998-pa) | TC014499 | 0.02224 | 1 |
| chitin-binding domain containing protein | TC004500 | 0.02192 | 2 |
| beta-n-acetylglucosaminidase nag2 | TC011540 | 0.02065 | 5 |
| cuticular protein 47ef cg13214-pa | TC002595 | 0.01994 | 2 |
| pupal cuticle protein | TC013809 | 0.01994 | 1 |
| cuticular protein rr-1 family (agap009876-pa) | TC013130 | 0.01944 | 1 |
| laccase-like multicopper oxidase 1 (TcLac2) | TC010489 | 0.01933 | 6 |
| beta nu integrin subunit (TcPSBI-3) | TC005782 | 0.01891 | 6 |
| cuticular protein 50cb | TC006981 | 0.0188 | 5 |
| chitin deacetylase 3 | TC005409 | 0.01828 | 4 |
| tpa: cuticle protein | TC015908 | 0.01752 | 1 |
| cuticle protein cp5 | TC014720 | 0.01739 | 1 |
| cuticular protein rr-2 family (agap001664-pa) | TC008768 | 0.0172 | 2 |
| cuticular protein rr-1 family (agap009876-pa) | TC014685 | 0.01701 | 1 |
| endocuticle structural glycoprotein bd- | TC014686 | 0.01652 | 1 |
| cuticular protein 51a | TC015720 | 0.01617 | 1 |
| cg34355 cg34355-pa (TcKNK2) | TC012301 | 0.01608 | 5 |
| cdc42 gtpase-activating protein (TcPMP1&2) | TC006098 | 0.01596 | 15 |
| cuticular protein rr-2 family (agap001669-pa) | TC003109 | 0.01584 | 1 |
| cuticular protein rr-1 family (agap009876-pa) | TC013128 | 0.01522 | 1 |
| adult cuticular protein | TC013828 | 0.01492 | 1 |
| chitinase 6 (chitinase 18) | TC009630 | 0.01445 | 2 |
| cysteine sulfinic acid (TcDc CG5618) | TC014177 | 0.01439 | 3 |
| brain chitinase and chia | TC003179 | 0.01385 | 1 |
| tpa: cuticle protein | TC003830 | 0.01384 | 2 |
| protein yellow (Tcyellow-2) | TC003539 | 0.01345 | 2 |
| chitinase (chitinase 5) | TC001770 | 0.01299 | 3 |
| cuticular protein 47ef cg13214-pa | TC003835 | 0.01299 | 1 |
| chitin deacetylase 1 (CDA8) | TC014147 | 0.01227 | 2 |
| cuticular protein 50cb | TC004010 | 0.0118 | 1 |
| chitinase 7 | TC015481 | 0.01174 | 5 |
| integrin beta-ps (TcPSBI-2) | TC013706 | 0.01171 | 4 |
| cuticular protein | TC000369 | 0.01134 | 1 |
| cuticular protein 47ef cg13214-pa | TC004075 | 0.01134 | 3 |
| tpa: cuticle protein | TC003832 | 0.01056 | 1 |
| chitin binding protein | TC013568 | 0.0101 | 2 |
| cuticle | TC000851 | 0.009926 | 1 |
| cuticular protein 62bc cg1919-pa | TC008767 | 0.009926 | 1 |
| cuticular protein analogous to peritrophins 1-g | TC008877 | 0.0098 | 1 |
| cuticular protein 47ef cg13214-pa | TC004546 | 0.00944 | 1 |
| pupal cuticle protein c1b (tm-c1b) (tm-pcp c1b) | TC002840 | 0.008964 | 1 |
| cuticle protein | TC010056 | 0.008964 | 1 |
| hypothetical protein TcasGA2_TC016350 | TC016350 | 0.008349 | 1 |
| tpa: cuticle protein | TC003831 | 0.006934 | 1 |
| inwardly rectifying k+ (Kir2) | TC001199 | 0.006723 | 1 |
| chitin deacetylase 1 (CDA7) | TC013661 | 0.006497 | 1 |
| peritrophic matrix protein 14 (TcPMP5) | TC003273 | 0.0062 | 1 |
| chitin deacetylase 1 (CDA6) | TC013662 | 0.005725 | 1 |
| adult cuticle | TC011338 | 0.005468 | 1 |
| cuticular protein cpg12 | TC006985 | 0.005305 | 2 |
| cuticular protein analogous to peritrophins 1-j | TC011101 | 0.005272 | 3 |
| ionotropic glutamate receptor-invertebrate | TC016300 | 0.004931 | 1 |
| chitinase 13 (chitinase 19) | TC009175 | 0.004798 | 1 |
| inwardly rectifying k+ (TcKir3) | TC006707 | 0.004228 | 1 |
| cuticular protein 144 (agap006369-pa) | TC016311 | 0.003569 | 1 |
| laccase-like multicopper oxidase 1 (TcLLP) | TC015880 | 0 | 0 |
| hexosaminidase isoform a (TcNAG1) | TC009808 | 0 | 0 |
| fused lobes (TcNAG3) | TC001116 | 0 | 0 |
| inwardly rectifying k+ (TcKir4) | TC006708 | 0 | 0 |
| aspartate 1-decarboxylase (TcADC2) | TC010581 | 0 | 0 |
| TcPMP6 | TC008506 | 0 | 0 |
| peritrophic matrix protein 2-b (TcPMP7) | TC003275 | 0 | 0 |
| yellow-g-like protein | TC006226 | 0 | 0 |
| protein yellow (Tcyellow-G2) | TC005927 | 0 | 0 |
| mucin-like protein (TcPMP3) | TC009232 | 0 | 0 |
| histidine decarboxylase | TC010062 | 0 | 0 |
| cuticular protein | TC000370 | 0 | 0 |
| cuticular protein | TC001177 | 0 | 0 |
| cuticular protein | TC001178 | 0 | 0 |
| cuticular protein rr-2 family (agap006261-pa) | TC002908 | 0 | 0 |
| cuticular protein rr-2 family (agap006828-pa) | TC003509 | 0 | 0 |
| cuticular protein 47ef cg13214-pa | TC004067 | 0 | 0 |
| cuticular protein 47ef cg13214-pa | TC004072 | 0 | 0 |
| cuticular protein 47ef cg13214-pa | TC004073 | 0 | 0 |
| cuticular protein 49aa cg30045-pb | TC004547 | 0 | 0 |
| cuticular protein rr-1 family (agap010887-pa) | TC004548 | 0 | 0 |
| cuticular protein 92f | TC006646 | 0 | 0 |
| cuticular protein rr-2 family (agap012466-pa) partial | TC006989 | 0 | 0 |
| cuticular protein | TC007240 | 0 | 0 |
| cuticular protein | TC007241 | 0 | 0 |
| cuticular protein | TC008227 | 0 | 0 |
| cuticular protein | TC008230 | 0 | 0 |
| cuticular protein rr-2 family (agap001664-pa) | TC008769 | 0 | 0 |
| cuticular protein 92a | TC008770 | 0 | 0 |
| cuticular protein | TC009873 | 0 | 0 |
| cuticular protein rr-2 family (agap001664-pa) | TC010057 | 0 | 0 |
| cuticular protein analogous to peritrophins 1-i | TC012766 | 0 | 0 |
| cuticular protein 47ef cg13214-pa | TC013127 | 0 | 0 |
| cuticular protein 49ab | TC013133 | 0 | 0 |
| chitinase 3 (chitinase 9) | TC009177 | 0 | 0 |
| chitinase 13 (chitinase 12) | TC009178 | 0 | 0 |
| chitinase 13 (chitinase 6) | TC009179 | 0 | 0 |
| chitinase 4 | TC009180 | 0 | 0 |
| teratocyte released chitinase (chitinase 8) | TC009624 | 0 | 0 |
| chitinase 6 (chitinase 17) | TC009625 | 0 | 0 |
| chitinase 13 (chitinase 2) | TC009626 | 0 | 0 |
| chitinase 13 (chitinase 11) | TC009627 | 0 | 0 |
| chitinase 13 (chtinase 13 or 14?) | TC009628 | 0 | 0 |
| chitinase 3 (chitinase 15) | TC009629 | 0 | 0 |
| larval cuticle protein a3a (tm-a3a) (tm-lcp a3a) | TC000721 | 0 | 0 |
| cuticle protein | TC000722 | 0 | 0 |
| larval cuticle protein a3a (tm-a3a) (tm-lcp a3a) | TC000725 | 0 | 0 |
| larval cuticle protein a3a (tm-a3a) (tm-lcp a3a) | TC000852 | 0 | 0 |
| tpa: cuticle protein | TC001121 | 0 | 0 |
| pupal cuticle protein c1b (tm-c1b) (tm-pcp c1b) | TC002841 | 0 | 0 |
| cuticle protein | TC003363 | 0 | 0 |
| pupal cuticle protein c1b (tm-c1b) (tm-pcp c1b) | TC003599 | 0 | 0 |
| tpa: cuticle protein | TC003834 | 0 | 0 |
| endocuticle structural glycoprotein bd-2 | TC005548 | 0 | 0 |
| cuticle protein | TC007724 | 0 | 0 |
| tpa: cuticle protein | TC008295 | 0 | 0 |
| cuticle protein 6 | TC008400 | 0 | 0 |
| cuticle protein 34 | TC011148 | 0 | 0 |
| pupal cuticle | TC011149 | 0 | 0 |
| pupal cuticle | TC011337 | 0 | 0 |
| pupal cuticle protein | TC013129 | 0 | 0 |
| cuticular protein 49ab | TC013133 | 0 | 0 |
| pupal cuticle | TC013306 | 0 | 0 |
| pupal cuticle | TC013307 | 0 | 0 |
| tpa: cuticle protein | TC013808 | 0 | 0 |
| cuticular protein rr-1 family (agap006283-pa) | TC013810 | 0 | 0 |
| cuticular protein 62bc cg1919-pa | TC013811 | 0 | 0 |
| tpa: cuticle protein | TC013814 | 0 | 0 |
| cuticular protein 62bc cg1919-pa | TC013817 | 0 | 0 |
| cuticular protein 62bc cg1919-pa | TC013818 | 0 | 0 |
| adult cuticle | TC013819 | 0 | 0 |
| adult cuticle | TC013820 | 0 | 0 |
| adult cuticle | TC013821 | 0 | 0 |
| adult cuticle | TC013822 | 0 | 0 |
| adult cuticle | TC013823 | 0 | 0 |
| adult cuticle | TC013824 | 0 | 0 |
| adult cuticle | TC013825 | 0 | 0 |
| adult cuticle | TC013826 | 0 | 0 |
| pupal cuticle protein | TC013827 | 0 | 0 |
| adult cuticle | TC013989 | 0 | 0 |
| cuticular protein 62bc cg1919-pa | TC013990 | 0 | 0 |
| adult cuticle | TC013992 | 0 | 0 |
| pupal cuticle | TC014497 | 0 | 0 |
| larval cuticle protein | TC014498 | 0 | 0 |
| pupal cuticle | TC014500 | 0 | 0 |
| endocuticle structural glycoprotein bd- | TC014770 | 0 | 0 |
| adult cuticle | TC015901 | 0 | 0 |
|  |  |  |  |

Table S3: Expression of immunity-related genes in the TcA cell line.

| Gene name | Gene family | TC# | RPKM | Reads |
| --- | --- | --- | --- | --- |
| PGRP-LD | PGRP | TC002546 | 0 | 0 |
| PGRP-LA | PGRP | TC002789 | 3.933 | 585 |
| PGRP-LC | PGRP | TC002790 | 2.398 | 394 |
| PGRP-LE | PGRP | TC010508 | 0.491 | 69 |
| PGRP-SA | PGRP | TC010611 | 2.053 | 174 |
| PGRP-SB | PGRP | TC013620 | 0.1591 | 13 |
| PGRP-LB | PGRP | TC015689 | 0.2557 | 23 |
| βGRP1 | βGRP/GNBP | TC002295 | 0.0122 | 2 |
| βGRP3 | βGRP/GNBP | TC003991 | 0.1679 | 35 |
| βGRP2 | βGRP/GNBP | TC011529 | 0.1727 | 33 |
| CTL1 | C-type lectin | TC006978 | 0.04257 | 6 |
| CTL2 | C-type lectin | TC014184 | 0.00756 | 1 |
| CTL3 | C-type lectin | TC010898 | 0.8264 | 144 |
| CTL4 | C-type lectin | TC010947 | 0.7892 | 101 |
| CTL5 | C-type lectin | TC010419 | 3.533 | 333 |
| CTL6 | C-type lectin | TC003708 | 0.03242 | 3 |
| CTL7 | C-type lectin | TC014053 | 0.1487 | 18 |
| CTL8 | C-type lectin | TC010412 | 0.00746 | 1 |
| CTL9 | C-type lectin | TC014328 | 0 | 0 |
| CTL10 | C-type lectin | TC003135 | 0.00819 | 2 |
| CTL11 | C-type lectin | TC003136 | 0.01706 | 15 |
| CTL12 | C-type lectin | TC013632 | 3.676 | 1877 |
| CTL13 | C-type lectin | TC013911 | 0.6879 | 69 |
| CTL14 | C-type lectin | TC000871 | 0.9157 | 1438 |
| CTL15 | C-type lectin | TC030667 | 5.207 | 394 |
|  |  | TC030754 | 0.8577 | 392 |
| CTL16 | C-type lectin | TC002984 | 0.1236 | 46 |
| GALE1 | galectin | TC007619 | 1.482 | 232 |
| GALE2 | galectin | TC011871 | 1.719 | 300 |
| GALE3 | galectin | TC014802 | 0.5352 | 286 |
| FREP5 | fibrinogen-like | TC003194 | 0.00486 | 1 |
| FREP1 | fibrinogen-like | TC003276 | 0 | 0 |
| FREP2 | fibrinogen-like | TC003277 | 0 | 0 |
| FREP3 | fibrinogen-like | TC003278 | 0 | 0 |
| FREP4 | fibrinogen-like | TC003294 | 0 | 0 |
| FREP6 | fibrinogen-like | TC004004 | 0.01086 | 3 |
| FREP7 | fibrinogen-like | TC004864 | 0 | 0 |
| TEP-B | TEP | TC014664 | 4.961 | 3166 |
| TEP-C | TEP | TC009667 | 0.00636 | 4 |
| TEP-A | TEP | TC009375 | 3.081 | 2354 |
| TEP-D | TEP | TC000808 | 0.00404 | 3 |
| H1 | cSPH | TC000246 | 0.3804 | 62 |
| H2 | cSPH | TC000247 | 10.41 | 1810 |
| H3 | cSPH | TC000248 | 2.063 | 363 |
| H4 | cSPH | TC000249 | 0.1466 | 27 |
| H5 | cSPH | TC000250 | 0 | 0 |
| H6 | cSPH | TC000252 | 1.253 | 639 |
| P7 | cSP | TC000494 | 0.01208 | 2 |
| P8 | cSP | TC000495 | 0.2597 | 42 |
| P9 | SP | TC000496 | 0 | 0 |
| P10 | cSP | TC000497 | 0 | 0 |
| P11 | SP | TC000545 | 0 | 0 |
| P12 | SP | TC000546 | 0 | 0 |
| P13 | SP | TC000547 | 0 | 0 |
| H14 | SPH | TC000548 | 0.00779 | 1 |
| P15 | SP | TC000550 | 0 | 0 |
| P16 | SP | TC000635 | 0.7388 | 92 |
| H17 | SPH | TC000740 | 0.3815 | 48 |
| H18 | SPH | TC000829 | 12.08 | 2063 |
| P19 | SP | TC000870 | 0.00282 | 2 |
| P20 | SP | TC030061 | 0 | 0 |
| P21 | SP | TC030062 | 0.02532 | 3 |
| P22 | SP | TC001023 | 0 | 0 |
| P23 | SP | TC001157 | 0.0085 | 1 |
| P24 | SP | TC030063 | 0 | 0 |
| P25 | SP | TC030064 | 0 | 0 |
| P26 | SP | TC030065 | 0 | 0 |
| P27 | SP | TC001159 | 0 | 0 |
| H28 | cSPH | TC001300 | 0 | 0 |
| H29 | cSPH | TC001301 | 0.03898 | 6 |
| H31 | SPH | TC001946 | 0.2612 | 34 |
| P32 | SP | TC002061 | 0.1641 | 21 |
| H33 | cSPH | TC002112 | 0 | 0 |
| H34 | cSPH | TC002150 | 0.01473 | 2 |
| H35 | cSPH | TC002193 | 0.00692 | 1 |
| P36 | SP | TC002659 | 0.1779 | 16 |
| P37 | SP | TC002766 | 0 | 0 |
| P38 | SP | TC002767 | 0 | 0 |
| P39 | SP | TC002768 | 0 | 0 |
| P40 | SP | TC002785 | 0 | 0 |
| P41 | SP | TC002786 | 0 | 0 |
| P42 | SP | TC003081 | 0.00753 | 1 |
| P43 | SP | TC004084 | 0 | 0 |
| P44 | cSP | TC004160 | 1.674 | 367 |
| P45 | SP | TC004418 | 0 | 0 |
| P46 | SP | TC004523 | 0.4485 | 126.8 |
| H47 | SPH | TC030066 | 0.3304 | 55 |
| H49 | SPH | TC030068 | 0.5531 | 77 |
| P50 | SP | TC004535 | 0.07986 | 22 |
| H51 | cSPH | TC004622 | 0.03818 | 12 |
| P52 | cSP | TC004624 | 0 | 0 |
| P53 | cSP | TC004635 | 0.1205 | 26 |
| P54 | SP | TC004654 | 0.01112 | 6 |
| P55 | cSP | TC004770 | 3.733 | 602 |
| P56 | cSP | TC004863 | 0.0065 | 1 |
| H57 | SPH | TC004900 | 0.01267 | 2 |
| P58 | SP | TC004937 | 0.7272 | 61 |
| H59 | cSPH | TC004957 | 0 | 0 |
| P60 | cSP | TC005130 | 0.00634 | 1 |
| P61 | cSP | TC005230 | 0.03248 | 5 |
| P62 | SP | TC005327 | 0.04854 | 14 |
| H63 | SPH | TC005635 | 0 | 0 |
| H64 | SPH | TC005908 | 0.1043 | 12 |
| H65 | SPH | TC005925 | 0 | 0 |
| P66 | cSP | TC005976 | 0.03076 | 5 |
| P67 | SP | TC006026 | 0.03887 | 4 |
| P68 | SP | TC006033 | 2.333 | 567 |
| P69 | SP | TC006034 | 8.308 | 1717 |
| H70 | SPH | TC006246 | 0.1742 | 18.98 |
| H71 | SPH | TC006247 | 0.0091 | 0.9989 |
| P72 | SP | TC006268 | 0.02523 | 3 |
| H73 | SPH | TC006269 | 0.0404 | 4 |
| P74 | SP | TC006424 | 0.01601 | 2 |
| P75 | SP | TC006438 | 0.00886 | 1 |
| P76 | SP | TC007017 | 0.00893 | 1 |
| P77 | SP | TC007019 | 0 | 0 |
| H78 | cSPH | TC007026 | 0 | 0 |
| P79 | SP | TC008267 | 0 | 0 |
| P80 | SP | TC008504 | 0 | 0 |
| H81 | SPH | TC008505 | 0 | 0 |
| H82 | cSPH | TC008554 | 0 | 0 |
| P83 | cSP | TC008653 | 0.00672 | 2 |
| P84 | cSP | TC008657 | 0 | 0 |
| H85 | cSPH | TC030609 | 0 | 0 |
| P86 | cSP | TC030609 | 0 | 0 |
| P87 | cSP | TC008659 | 0 | 0 |
| H88 | SPH | TC008930 | 0 | 0 |
| H89 | SPH | TC008931 | 0 | 0 |
| P90 | cSP | TC009089 | 0.2052 | 33 |
| P91 | cSP | TC009090 | 1.509 | 257 |
| P92 | cSP | TC009091 | 0 | 0 |
| P93 | cSP | TC009092 | 0.0123 | 2 |
| P94 | cSP | TC009093 | 0 | 0 |
| P95 | cSP | TC009094 | 0.00641 | 1 |
| P96 | SP | TC009602 | 0 | 0 |
| P98 | SP | TC030073 | 0.00573 | 1 |
| H99 | cSPH | TC010076 | 0.8524 | 136 |
| P100 | SP | TC010781 | 0 | 0 |
| H101 | SPH | TC010904 | 0 | 0 |
| H102 | SPH | TC030074 | 0 | 0 |
| H103 | SPH | TC030075 | 0 | 0 |
| H104 | cSPH | TC010906 | 0 | 0 |
| H105 | SPH | TC010907 | 0 | 0 |
| H106 | SPH | TC010908 | 0 | 0 |
| H107 | SPH | TC010909 | 0 | 0 |
| H108 | SPH | TC010910 | 0 | 0 |
| H109 | SPH | TC010911 | 0 | 0 |
| H110 | SPH | TC010927 | 0.0087 | 1 |
| H111 | SPH | TC010929 | 0 | 0 |
| H112 | SPH | TC010930 | 0 | 0 |
| H113 | SPH | TC010932 | 0 | 0 |
| H114 | SPH | TC010933 | 0 | 0 |
| H115 | SPH | TC010934 | 0 | 0 |
| H116 | SPH | TC010935 | 0 | 0 |
| H117 | SPH | TC010936 | 0 | 0 |
| H118 | SPH | TC010937 | 0 | 0 |
| H119 | SPH | TC010938 | 0 | 0 |
| H120 | SPH | TC010939 | 0 | 0 |
| P121 | SP | TC010940 | 0 | 0 |
| H122 | SPH | TC010941 | 0.00893 | 1 |
| P123 | SP | TC010959 | 0 | 0 |
| P124 | SP | TC011014 | 0.009 | 1 |
| H125 | cSPH | TC011067 | 0 | 0 |
| P126 | cSP | TC011078 | 0.2281 | 45 |
| P127 | SP | TC011824 | 0 | 0 |
| P128 | SP | TC011825 | 0 | 0 |
| H129 | SPH | TC012390 | 0.2303 | 72 |
| H130 | SPH | TC012573 | 0.04347 | 5 |
| H131 | SPH | TC012574 | 0.01786 | 2 |
| H132 | SPH | TC012575 | 1.45 | 173 |
| P133 | SP | TC013042 | 0.0054 | 1 |
| P134 | SP | TC013084 | 0 | 0 |
| P135 | SP | TC013276 | 0.00781 | 1 |
| P136 | cSP | TC013277 | 8.29 | 1380 |
| H137 | cSPH | TC013278 | 0.01373 | 2 |
| P138 | cSP | TC013279 | 0 | 0 |
| P139 | SP | TC013280 | 2.039 | 268 |
| P140 | cSP | TC013326 | 0.2445 | 37 |
| P141 | SP | TC013415 | 0.6746 | 84 |
| P142 | cSP | TC013416 | 0.01971 | 3 |
| H143 | SPH | TC013421 | 0 | 0 |
| P144 | SP | TC013613 | 0.00548 | 1 |
| P145 | SP | TC013709 | 0 | 0 |
| H146 | SPH | TC013894 | 3.797 | 3502 |
| P147 | SP | TC014083 | 0 | 0 |
| H148 | SPH | TC014375 | 0 | 0 |
| P149 | SP | TC014391 | 0.0086 | 1 |
| P150 | SP | TC014930 | 0 | 0 |
| P151 | SP | TC015083 | 0.05761 | 7 |
| H152 | SPH | TC015099 | 0 | 0 |
| P153 | SP | TC015110 | 0.00875 | 3 |
| P154 | SP | TC015130 | 0.07144 | 8 |
| P155 | SP | TC015237 | 0 | 0 |
| P156 | SP | TC015295 | 0.01986 | 7 |
| P157 | SP | TC015297 | 0.02558 | 5 |
| H158 | SPH | TC015344 | 0 | 0 |
| H159 | SPH | TC015390 | 0 | 0 |
| P160 | SP | TC015579 | 0.04331 | 5 |
| P161 | SP | TC015580 | 0.02689 | 3 |
| P162 | SP | TC015617 | 0 | 0 |
| P163 | SP | TC015618 | 0 | 0 |
| H164 | cSPH | TC015670 | 0.2239 | 67 |
| P165 | SP | TC015779 | 0 | 0 |
| P166 | SP | TC015780 | 0.00841 | 1 |
| P167 | SP | TC016121 | 0.0094 | 1 |
| P168 | SP | TC016372 | 0 | 0 |
| serpin1 | serpin | TC000760 | 25.08 | 3861 |
| serpin2 | serpin | TC002085 | 0.03534 | 18 |
| serpin3 | serpin | TC002247 | 1.448 | 256 |
| serpin4 | serpin | TC030076 | 3.573 | 689 |
| serpin5 | serpin | TC030077 | 2.443 | 430 |
| serpin6 | serpin | TC005065 | 0.06503 | 16 |
| serpin7 | serpin | TC005740 | 3.966 | 679 |
| serpin8 | serpin | TC005741 | 0.02403 | 4 |
| serpin10 | serpin | TC005742 | 0.00893 | 1 |
| serpin11 | serpin | TC005743 | 0.0073 | 1 |
| serpin12 | serpin | TC005744 | 0 | 0 |
| serpin13 | serpin | TC030079 | 0 | 0 |
| serpin14 | serpin | TC030080 | 0 | 0 |
| serpin15 | serpin | TC005746 | 0 | 0 |
| serpin16 | serpin | TC005747 | 0 | 0 |
| serpin17 | serpin | TC005749 | 0 | 0 |
| serpin18 | serpin | TC005750 | 0.1517 | 25.97 |
| serpin19 | serpin | TC005751 | 0.3892 | 66.81 |
| serpin20 | serpin | TC005752 | 0.8893 | 151.9 |
| serpin21 | serpin | TC005753 | 0.4048 | 79.98 |
| serpin22 | serpin | TC005754 | 0.134 | 23 |
| serpin23 | serpin | TC005771 | 0.02397 | 4 |
| serpin24 | serpin | TC006255 | 0.1183 | 20 |
| serpin25 | serpin | TC006607 | 0 | 0 |
| serpin26 | serpin | TC007869 | 1.371 | 345 |
| serpin27 | serpin | TC011718 | 1.513 | 327 |
| serpin28 | serpin | TC013310 | 16.84 | 3669 |
| serpin29 | serpin | TC013389 | 1.576 | 304 |
| serpin30 | serpin | TC014237 | 4.132 | 720 |
| serpin31 | serpin | TC015224 | 0.01208 | 2 |
| spz1 | spätzle | TC000520 | 1.359 | 134 |
| spz7 | spätzle | TC001053 | 0.01257 | 1 |
| spz2 | spätzle | TC001054 | 0.3429 | 41.96 |
| spz3 | spätzle | TC030608 | 1.001 | 135 |
|  |  | TC030639 | 0.7235 | 61 |
| spz4 | spätzle | TC006726 | 0 | 0 |
| spz5 | spätzle | TC013304 | 0 | 0 |
| spz6 | spätzle | TC016368 | 0.01123 | 2 |
| Toll9 | Toll-like receptor | TC000625 | 0.1258 | 74 |
| Toll1 | Toll-like receptor | TC000176 | 0.0116 | 3 |
| Toll3 | Toll-like receptor | TC004438 | 2.467 | 1117 |
| Toll4 | Toll-like receptor | TC004439 | 0.07407 | 27.99 |
| Toll2 | Toll-like receptor | TC004452 | 0.00768 | 3 |
| Toll7 | Toll-like receptor | TC004474 | 2.329 | 1320 |
| Toll6 | Toll-like receptor | TC004895 | 0.02725 | 15 |
| Toll8 | Toll-like receptor | TC004898 | 0.6483 | 340 |
| Toll10 | Toll-like receptor | TC004901 | 0.1644 | 94 |
| ML1 | MD2-like | TC008202 | 1.707 | 107 |
| ML2 | MD2-like | TC008203 | 0 | 0 |
| ML3 | MD2-like | TC014068 | 0.01512 | 1 |
| ML4 | MD2-like | TC014069 | 0.01532 | 1 |
| ML5 | MD2-like | TC016351 | 1.135 | 79 |
| ML6 | MD2-like | TC007252 | 0.02928 | 2 |
| ML7 | MD2-like | TC014067 | 0 | 0 |
| ML8 | MD2-like | TC016352 | 0.1031 | 7 |
| cactus | cactus | TC002003 | 2.173 | 342 |
| pelle | pelle | TC015365 | 0.2709 | 52 |
| Myd88 | Myd88 | TC003185 | 1.009 | 175 |
| Tube | Tube | TC011895 | 0.6931 | 193 |
| pellino | pellino | TC009672 | 1.966 | 397 |
| Traf2 | Traf | TC007706 | 1.038 | 179 |
| cactin | cactin | TC008782 | 0.7151 | 329 |
| Dif1 | REL | TC007697 | 2.375 | 728 |
| Dif2 | REL | TC008096 | 0.9852 | 164 |
| FADD | FADD | TC014042 | 0.3658 | 31 |
| IKKb | IKKb | TC001419 | 0.2231 | 67.99 |
| IKKb | IKKb | TC009798 | 1.745 | 553 |
| IKKg | IKKg | TC000541 | 1.276 | 278 |
| IMD | IMD | TC010851 | 0.6013 | 52 |
| TAK1 | TAK | TC005572 | 0.5737 | 127 |
| casps1 | caspase | TC003841 | 0.3954 | 53 |
| casps2 | caspase | TC012581 | 0.338 | 57 |
| casps3 | caspase | TC012580 | 0.7709 | 132 |
| casps4 | dredd/casp8 | TC014026 | 0.756 | 186 |
| casps5 | caspase | TC000105 | 0.00727 | 1 |
| casps6 | caspase | TC012579 | 0.1836 | 32 |
| casps7 | caspase | TC002397 | 0.6385 | 39.75 |
| casps8 | caspase | TC000068 | 0 | 0 |
| caspar | blocking caspase | TC009985 | 0.6829 | 194 |
| IAP2 | IAP | TC001189 | 1.934 | 414 |
| IAP1 | IAP | TC001192 | 4.837 | 709 |
| IAP3 | IAP | TC009848 | 1.629 | 3054 |
| IAP4 | IAP | TC002709 | 0.5984 | 37 |
| REL1 | REL | TC011191 | 2.687 | 992 |
| REL2 | REL | TC014708 | 2.489 | 1129 |
| Tab2 | Tab2 | TC005952 | 0.4862 | 111 |
| Hep | Hep | TC000385 | 0.4439 | 124 |
| basket1 | basket | TC006810 | 0.7394 | 125 |
| basket2 | basket | TC011967 | 0.4588 | 74 |
| basket3 | basket | TC013594 | 2.064 | 315 |
| Jra | Jra | TC006814 | 2.353 | 232 |
| kay | kay | TC011870 | 6.365 | 1010 |
| DOME | DOME | TC001874 | 3.056 | 1455 |
| HOP | HOP | TC008648 | 0.5002 | 109 |
| STAT | STAT | TC013218 | 3.5 | 1182 |
| proPO1 | prophenoloxidase | TC000325 | 0.01354 | 4 |
| proPO2 | prophenoloxidase | TC014907 | 0.01351 | 3.994 |
| proPO3 | prophenoloxidase | TC015848 | 0.04163 | 5.994 |
| MI | melanization inhibitor | TC006342 | 0.00652 | 1 |
| hexamerin1 | hexamerin | TC005374 | 0 | 0 |
| hexamerin2 | hexamerin | TC005375 | 0 | 0 |
| hexamerin3 | hexamerin | TC005376 | 0 | 0 |
| hexamerin4 | hexamerin | TC005377 | 0 | 0 |
| hexamerin5 | hexamerin | TC006515 | 0.1873 | 57 |
| hexamerin6 | hexamerin | TC006769 | 0 | 0 |
| catalase1 | catalase | TC011385 | 0 | 0 |
| catalase2 | catalase | TC011090 | 0.00964 | 2 |
| GTX1 | glutathione oxidase | TC010362 | 1.156 | 100 |
| GTX2 | glutathione oxidase | TC010355 | 10.52 | 769 |
| GTX3 | glutathione oxidase | TC010354 | 0.3835 | 33 |
| HPX1 | heme peroxidase | TC005493 | 0.6148 | 390 |
| HPX2 | heme peroxidase | TC015234 | 0 | 0 |
| HPX3 | heme peroxidase | TC011222 | 0.08187 | 32 |
| HPX4 | heme peroxidase | TC004579 | 0.1915 | 64 |
| HPX5 | heme peroxidase | TC004551 | 2.905 | 932 |
| HPX6 | heme peroxidase | TC000751 | 0 | 0 |
| HPX7 | heme peroxidase | TC000175 | 0.03775 | 11 |
| HPX8 | heme peroxidase | TC004661 | 0.04978 | 13 |
| HPX9 | heme peroxidase | TC001556 | 0.333 | 200 |
| HPX10 | heme peroxidase | TC002498 | 0.2029 | 133 |
| HPX11 | heme peroxidase | TC004592 | 0.00486 | 2 |
| TPX6 | peroxiredoxin | TC014929 | 10.3 | 877 |
| TPX2 | peroxiredoxin | TC001700 | 0.04696 | 4 |
| TPX1 | peroxiredoxin | TC012328 | 0.7215 | 73 |
| TPX3 | peroxiredoxin | TC001071 | 1.839 | 194 |
| TPX5 | peroxiredoxin | TC013791 | 0.3259 | 31 |
| TPX4 | peroxiredoxin | TC004948 | 0.02065 | 2 |
| SOD3 | superoxide dismutase | TC007011 | 11.67 | 777 |
| SOD2 | superoxide dismutase | TC011676 | 48.72 | 3518 |
| SOD4 | superoxide dismutase | TC011675 | 2.932 | 289 |
| SOD1 | superoxide dismutase | TC011770 | 0.5553 | 267 |
| attacin1 | antimicrobial peptide | TC007737 | 0.1115 | 8 |
| attacin2 | antimicrobial peptide | TC007738 | 0.1267 | 8 |
| attacin3 | antimicrobial peptide | TC007739 | 0.01552 | 1 |
| cecropin1 | antimicrobial peptide | |  |  |
| cecropin2 | antimicrobial peptide | TC030482 | 0 | 0 |
| cecropin3 | antimicrobial peptide | TC000500 | 0.02542 | 1 |
| defensin1 | antimicrobial peptide | TC006250 | 0.03476 | 1.999 |
| defensin2 | antimicrobial peptide | TC010517 | 0.05782 | 2 |
| defensin3 | antimicrobial peptide | TC012469 | 3.634 | 132 |
| defensin4 | antimicrobial peptide | |  |  |
| coleoptericin1 | antimicrobial peptide | TC005093 | 0 | 0 |
| coleoptericin2 | antimicrobial peptide | TC005096 | 0.01626 | 0.9986 |
| lysozyme1 | lysozyme | TC010349 | 0.00838 | 1 |
| lysozyme2 | lysozyme | TC010350 | 0 | 0 |
| lysozyme3 | lysozyme | TC010351 | 0 | 0 |
| lysozyme4 | lysozyme | TC010352 | 0 | 0 |
| WAP | antimicrobial peptide | TC011324 | 0.07521 | 4 |
| neuroglian | neuroglian/hemolin | TC001889 | 7.538 | 4133 |
| SR-B6 | scavenger receptor | TC000948 | 0.01745 | 4 |
| SR-B7 | scavenger receptor | TC007247 | 2.442 | 490 |
| SR-A1 | scavenger receptor | TC007861 | 1.826 | 2364 |
| SR-B10 | scavenger receptor | TC008191 | 0.00446 | 1 |
| SR-B1 | scavenger receptor | TC008209 | 0.05389 | 12 |
| SR-B3 | scavenger receptor | TC008210 | 0.06065 | 37 |
| SR-B11 | scavenger receptor | TC010348 | 0 | 0 |
| SR-B12 | scavenger receptor | TC010353 | 0 | 0 |
| SR-B13 | scavenger receptor | TC010356 | 0.2123 | 113.9 |
| SR-A2 | scavenger receptor | TC011653 | 0.03839 | 8 |
| SR-B14 | scavenger receptor | TC012756 | 0 | 0 |
| SR-B15 | scavenger receptor | TC012757 | 0 | 0 |
| SR-B16 | scavenger receptor | TC012758 | 0 | 0 |
| SR-A3 | scavenger receptor | TC013894 | 3.797 | 3502 |
| SR-B5 | scavenger receptor | TC014946 | 0.1704 | 42 |
| SR-B8 | scavenger receptor | TC014951 | 0.1133 | 27 |
| SR-B9 | scavenger receptor | TC014954 | 5.154 | 1150 |
| SR-A4 | scavenger receptor | TC015110 | 0.00875 | 3 |
| SR-B4 | scavenger receptor | TC015144 | 0.04642 | 11 |
| SR-C | scavenger receptor | TC015640 | 0.08078 | 19 |
| SR-B2 | scavenger receptor | TC015854 | 0.00417 | 1 |
| NimA | nimrod | TC011427 | 0.01556 | 3 |
| NimB | nimrod | TC011428 | 8.335 | 1283 |
| NimCl1 | nimrod | TC002053 | 0 | 0 |
| NimCl2 | nimrod | TC015258 | 0 | 0 |
| draper | draper | TC000689 | 2.257 | 970 |

Figure S1.

The map for the plasmid used in this study. The sequence is deposited in NCBI with the GenBank Accession ############.


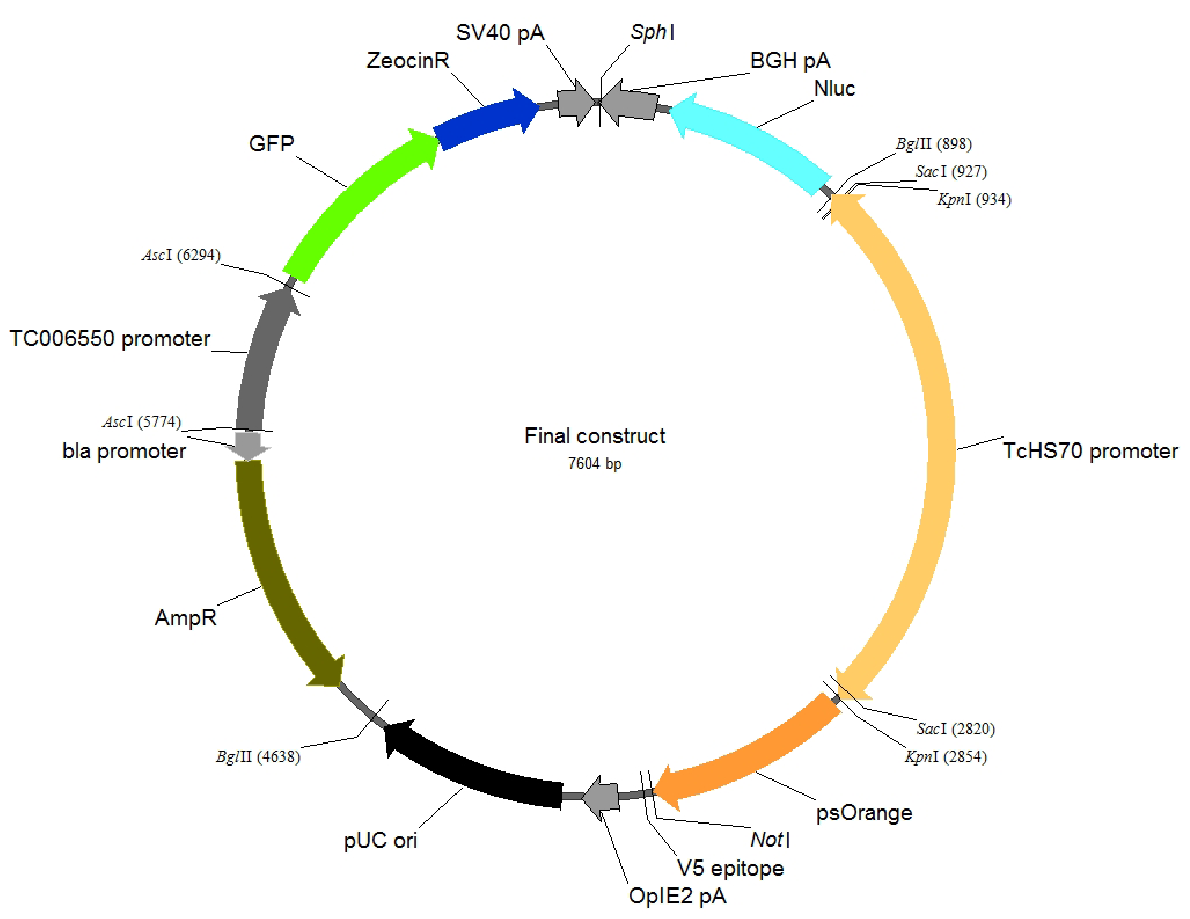

Supplement: Supplementary Information — Supplementary data [file srep06840-s1.docx]
